# Exploring the Effects of Osteopathic Manipulative Treatment on Autonomic Function Through the Lens of Heart Rate Variability

**DOI:** 10.3389/fnins.2020.579365

**Published:** 2020-10-07

**Authors:** Luca Carnevali, Luca Lombardi, Mauro Fornari, Andrea Sgoifo

**Affiliations:** ^1^Stress Physiology Lab, Department of Chemistry, Life Sciences and Environmental Sustainability, University of Parma, Parma, Italy; ^2^Stress Control Lab, Collegio Italiano di Osteopatia, Parma, Italy

**Keywords:** osteopathy, heart rate variability, autonomic, hypertension, stress, fatigue status

## Abstract

The osteopathic community has long hypothesized that the autonomic nervous system (ANS) represents one of the putative substrates through which osteopathic manipulative treatment (OMT) can improve body functions that have been altered by musculoskeletal alterations. Heart rate variability (HRV) is an important physiological measure of cardiac ANS activity. Emerging evidence suggests that OMT is associated with HRV changes that (i) are indicative of a larger cardiac vagal modulation, (ii) are independent from the part of the body needing treatment, (iii) occur even in the absence of musculoskeletal alterations. Yet, many questions remain unanswered, the duration of these effects and the specificity of HRV responses to different OMT techniques being perhaps the most critical. Therefore, this paper discusses prospects for future applications of HRV for the study of the influence of OMT on ANS function. Moreover, based on existing studies and preliminary data on the effects of OMT on HRV in specific pathological (hypertension) and physiological (stress exposure and recovery from sport competition) conditions that are commonly associated with increased sympathetic and/or decreased vagal activity, we propose that HRV analysis could be exploited to evaluate the effectiveness of OMT as a preventive or complementary strategy in clinical and non-clinical conditions characterized by ANS imbalance.

## Introduction

Osteopathic manipulative treatment (OMT), a non-invasive form of manual therapy, has evolved as a therapeutic approach aimed at correcting alterations in musculoskeletal structures that are having direct or indirect negative effects on the perfusion of body tissues and inherent physiological function. This perspective article is centered around the belief, within the osteopathic community, that the autonomic nervous system (ANS) represents one of the putative substrates of the action of OMT and its favorable effects on body functions (Henley et al., 2008; Rechberger et al., 2019). Somatic dysfunctions—which in the osteopathic terminology imply “impaired or altered function of related components of the body framework system: skeletal, arthrodial and myofascial structures, and related vascular, lymphatic and neural elements” (Glossary Review Committee, for the Educational Council on Osteopathic Principles and the American Association of Colleges of Osteopathic Medicine, 2017)—are treated using a wide variety of manual techniques, alone or in combination. The description of these techniques, which include articular and myofascial techniques, balanced ligamentous tension and craniosacral techniques, just to name a few, falls beyond the scope of this paper and can be found elsewhere (e.g., Campbell et al., 2012). Here, the focus is directed to the theoretical association between OMT and ANS, which is being supported by emerging empirical evidence obtained through physiological measures of ANS activity (Rechberger et al., 2019). Specifically, in the next sections we will discuss studies that have adopted heart rate variability (HRV) measures to evaluate the relationship between OMT and ANS activity in healthy subjects and in specific pathological and physiological conditions characterized by ANS imbalance, and we will offer new prospects for future applications of HRV for the study of the influence of OMT on ANS function.

## Autonomic Function and HRV

Autonomic regulation of most visceral organs reflects the balance between sympathetic and parasympathetic (vagal) modulation. This is particularly apparent in the neural control of the heart, where the balance between sympathetic excitation and vagal inhibition of sinoatrial node activity contributes to beat-to-beat heart rate fluctuations, also known as HRV. Traditionally, several HRV indices and methods have been used to quantify cardiac sympathetic and vagal influences (Task Force of the European Society of Cardiology and the North American Society of Pacing and Electrophysiology, 1996; Berntson et al., 1997; Shaffer and Ginsberg, 2017). However, it is now widely recognized that the association between ANS balance and HRV is non-trivial, particularly in light of the non-linear and non-reciprocal relationship between sympathetic and vagal activity, and caution has been advised with loose interpretations of HRV metrics (Goldstein et al., 2011; Billman, 2013; Reyes Del Paso et al., 2013; Laborde et al., 2017). While we do not want to dig into the intricacies of HRV research, it is worth mentioning for the purposes of this paper that measures of HRV that reflect fast changes are reliably interpreted as surrogate indexes of cardiac vagal function (Laborde et al., 2017). Commonly reported measures of vagally mediated HRV include the root mean square of successive beat-to-beat interval differences (RMSSD)—a time-domain measure—and the high frequency HRV (HF-HRV). Moreover, the low frequency (LF) to HF ratio (LF/HF) was long considered as representing the sympathovagal balance, although this stance has been highly criticized (e.g., Billman, 2013). In fact, given that the LF band does not reflect sympathetic activity (Goldstein et al., 2011; Rahman et al., 2011; Heathers, 2012), there is now a consensus to say that the precise physiological underpinnings of the LF/HF ratio are still unclear, making the interpretation of LF/HF data problematic at best (Laborde et al., 2017).

The use of HRV as a proxy for ANS function has become a popular approach in several clinical and investigational domains (e.g., Thayer et al., 2010, 2012; Kemp and Quintana, 2013). In fact, an optimal level of vagally mediated HRV is generally associated with health, self-regulatory capacity, and adaptability or resilience, whereas low vagally mediated HRV is considered a risk factor for adverse physical and psychological health outcomes (Thayer et al., 2012; Beauchaine and Thayer, 2015; Sgoifo et al., 2015; Williams et al., 2015; Carnevali et al., 2018). More recently, HRV analysis has also captured the attention of manual therapy research in an attempt to understand how different manual approaches can influence ANS function, including, as we will detail below, OMT.

## OMT and HRV in Healthy Subjects

To the best of our knowledge, the first investigation of the link between OMT and HRV dates back to 2008 (Henley et al., 2008). This crossover study employed one particular type of OMT technique, cervical myofascial release, and explored its effect on ECG-based HRV responses to a passive 50° head-up tilt test in a small group of healthy male and female subjects (19–50 years old). The OMT technique was administered for 2 min after the beginning of the tilt test and was compared with sham manipulation and no-touch control. Results indicated that OMT was associated with higher HF-HRV values and a lower LF/HF ratio during the last 5 min of the tilt test, thus providing the first quantitative demonstration of the potential influence of OMT on reactivity measures of HRV during passive standing. This was followed by the investigation of the effects of a single session of OMT on ECG-based resting measures of HRV in small samples of healthy male and female subjects (22–32 years old) (Shi et al., 2011; Giles et al., 2013). An increase in vagally mediated HRV (i.e., HF-HRV) compared to baseline was observed during 4 min of cranial OMT—with no differences compared with sham therapy (Shi et al., 2011)—and specifically during the last 6 min of a 15 min cervical OMT protocol compared with sham therapy and time control in a crossover design (Giles et al., 2013). Notably, the latter effect was reported in healthy subjects free of injury or somatic dysfunction and therefore cannot be attributed to the correction of underlying musculoskeletal conditions. Moreover, the increase in vagally mediated HRV during OMT was not associated with concomitant changes in respiratory rate. This is relevant in light of the known influence of respiratory rate on cardiac vagal tone and particularly on the HF-HRV component (Berntson et al., 1997; Hill and Siebenbrock, 2009; Laborde et al., 2017). To improve the clinical generalizability of these findings, a subsequent sham-controlled crossover study adopted different OMT techniques to correct specific somatic dysfunctions found on structural evaluation—instead of a pre-determined OMT technique—in a healthy sample (*n* = 57) of asymptomatic adults (18–35 years old, 51% males). Interestingly, an increase in measures of vagally mediated HRV obtained via plethysmography was observed during both the 15-min OMT treatment and the following 5 min when no hand contact was provided (Ruffini et al., 2015). These results were recently replicated by the same group (Cerritelli et al., 2020a), and appear consistent with HRV responses to other forms of manual therapy, including spinal manipulative therapy and manual cranial therapy, both in asymptomatic adults (Budgell and Hirano, 2001; Budgell and Polus, 2006; Welch and Boone, 2008) and children (Bayo-Tallon et al., 2019). Together, these studies provide preliminary evidence that manual therapy techniques, and OMT in particular for our purposes, are associated with HRV changes which seem to: (i) be indicative of a larger cardiac vagal modulation under several conditions (i.e., resting and passive standing), (ii) be independent from the part of the body needing treatment, (iii) occur even in the absence of somatic dysfunctions, and (iv) be evident in different age and sex groups. In our view, one major limitation of these studies is that HRV measures were obtained during or immediately after a single-session treatment, and therefore it is not possible to estimate the extent to which OMT-associated increases in vagally mediated HRV can endure over time. Moreover, considering that there are many types of OMT techniques, it would be interesting to test whether they are associated with different HRV responses in the same individual.

## OMT and HRV in Pathological and Physiological Conditions Characterized by ANS Imbalance

In light of the above reported HRV responses in healthy individuals, the use of OMT in conditions characterized by ANS imbalance seems intuitive, yet little quantitative data currently supports its effectiveness. Here, we present preliminary evidence of the effects of OMT on HRV in specific pathological (hypertension) and physiological (stress exposure and recovery from sport competition) conditions that are generally associated with increased sympathetic and/or decreased vagal activity.

### Hypertension

**FIGURE 1:**
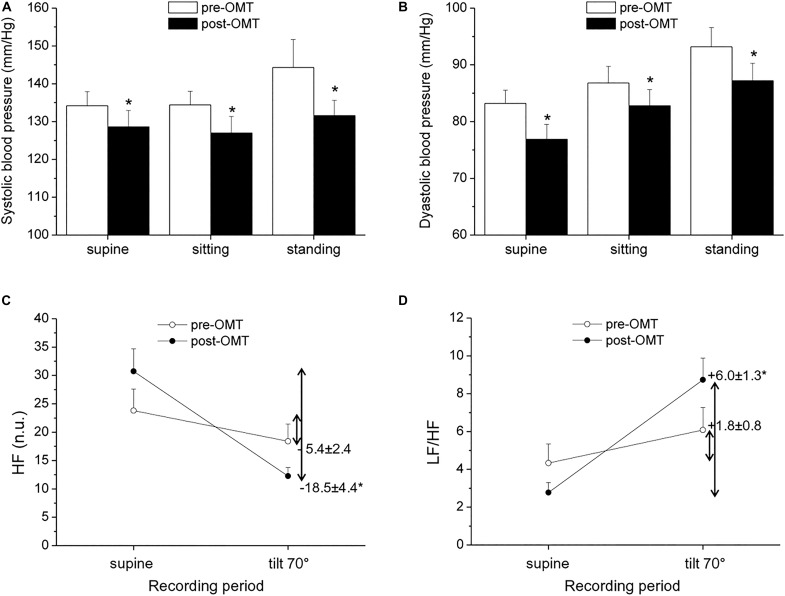
Pilot study conducted on 10 men with a confirmed diagnosis of hypertension [age: 54 ± 2 years; body mass index = 25.8 ± 1.0 kg/m^2^; non-smokers; no other clinical conditions; under regular antihypertensive treatment with calcium channel blockers (*n* = 6) and ACE inhibitors (*n* = 4)]. Blood pressure (BP) and heart rate variability (HRV) measures were obtained prior to and 48 h after an osteopathic manipulative treatment (OMT) protocol, consisting of four sessions of OMT, once a week for 4 weeks. Systolic **(A)** and diastolic **(B)** BP were measured with an electronic sphygmomanometer (A&D Medical: Model UA-631 V). High-frequency (HF, **C**) and low frequency (LF) /HF **(D)** data of HRV were calculated from ECG signals recorded with a BT16Plus device (Francesco Marazza Hardware & Software). ECG recordings lasted for 10 min in a supine position on a motorized table and during passive head-up tilting at 70°. Inner numbers in **(C,D)** represent delta changes (i.e., tilt value—supine value). Values are expressed as mean ± SEM. *indicates a significant difference (paired *t*-test *p* < 0.05).

Hypertension is commonly associated with increased sympathetic and decreased vagal activity (Julius, 1991), and reduced HRV has been described in hypertensive patients compared to normotensive individuals (Huikuri et al., 1996; Singh et al., 1998). In a non-randomized exploratory trial in which hypertensive subjects underwent OMT every fortnight for a period of 1 year, alongside routine hypertensive treatment, the authors found preliminary evidence of an association between OMT and reduced systolic BP (SBP), but not diastolic BP (DBP), compared to a control condition (Cerritelli et al., 2011). However, ANS function was not investigated in this study. More recently, BP and HRV were assessed in hypertensive and normotensive men aged 40–60 years before and after a single-session cranial OMT (Curi et al., 2018). Analysis of BP and HRV (derived from a cardiac monitor) revealed a decrease in SBP and DBP in hypertensive, but not normotensive, subjects, and a modest increase in vagally mediated HRV in both groups during the 15 min that followed OMT (Curi et al., 2018). However, as the authors acknowledged, the absence of a sham intervention and the short HRV and BP monitoring period after OMT limited the interpretation of these results. In a pilot and non-controlled study conducted in our lab, we evaluated BP and HRV in a small sample of hypertensive men undergoing four sessions of OMT, once a week for 4 weeks. The OMT intervention consisted of cervical myofascial release to improve vagus nerve function (Magoun, 1976; Henley et al., 2008), thoracic myofascial release and rib raising/articulation techniques to inhibit the sympathetic ganglionic chain, and lymphatic drainage techniques to contribute to body fluid homeostasis. BP and HRV assessment was conducted before the beginning of the OMT protocol and 48 h after the last OMT session, both at rest and in response to a passive 70° head-up tilt test. Our preliminary results indicate the presence of reduced resting values of SBP and DBP 48 h after the completion of the OMT protocol (Figures 1A,B). Moreover, we observed a larger reduction in HF-HRV values (Figure 1C) and a larger increase in the LF/HF ratio (Figure 1D) in response to passive standing compared with the pre-treatment assessment. Notably, previous studies have indicated that the reflex increase in sympathetic activity during passive standing is impaired in hypertension, probably because the sympathetic system is already hyperactive (Piccirillo et al., 1996; Heiskanen et al., 2011). Thus, keeping the limitations of the LF/HF in mind, our HRV data might hint at an improvement in cardiac ANS reactivity following OMT in hypertensive subjects. We believe that these results, although limited by their exploratory nature and clear methodological shortcomings, warrant new investigations involving randomized controlled trials on the effectiveness of OMT as a preventive and/or complementary treatment for hypertension and on the underlying ANS mechanisms.

### Stress Exposure

It is widely known that stressors tip the ANS balance toward a larger sympathetic prevalence and activate the hypothalamic-pituitary-adrenal axis (i.e., increase cortisol levels). In a between-subject study conducted by our group, a single-session of OMT using craniosacral techniques was performed immediately after an acute mental stressor on a small sample of healthy young men (20–30 years old) (Fornari et al., 2017). We found a smaller reduction in HF-HRV values (obtained from ECG analysis) and a much lower cortisol increment in response to the mental stressor in the OMT group compared with sham treatment (Fornari et al., 2017). While these results must be interpreted with caution given the exploratory nature of this study, in our view the association between OMT and reduced cardiac vagal withdrawal and cortisol rise in response to an acute stress represents an intriguing health-related outcome. In fact, building on the neurovisceral integration model (Thayer and Lane, 2000, 2009; Smith et al., 2017), sustained ANS imbalance is conceived as one feature of the biology of chronic stress that contributes to the development and progression of stress-related disorders. Relatedly, there is extensive evidence of reduced vagally mediated HRV in a number of stress-related emotional dysregulations, psychological disorders, and physical dysfunctions (Thayer et al., 2010, 2012; Beauchaine and Thayer, 2015; Sgoifo et al., 2015; Williams et al., 2015). Therefore, further investigation into the effects of OMT on HRV responses to acute and chronic stress conditions will provide novel insights into the potential utility of OMT as a preventive or complementary strategy in clinical and non-clinical settings associated with life stress (e.g., Dixon et al., 2020).

### Recovery From Sport Competition

Conditions of ANS imbalance characterized by decreased vagally mediated HRV have also been described in athletes in the aftermath of a sport competition or in the presence of overtraining syndrome (Iellamo et al., 2002; Mourot et al., 2004; Gratze et al., 2005; Bricout et al., 2010; Hellard et al., 2011; Boullosa et al., 2012; Edmonds et al., 2013). Consistent with this literature, in a recent study conducted by our group we found signs of reduced vagally mediated HRV (RMSSD and HF-HRV obtained from ECG analysis) and elevated mean arterial pressure 18–20 h after a rugby match in male players (Carnevali et al., 2020). Remarkably, in this sham-controlled crossover trial we showed that signs of cardiac vagal withdrawal and elevated mean arterial pressure were corrected by a players’ need-based OMT treatment addressing specific somatic dysfunctions found on structural evaluation (Carnevali et al., 2020). Previous studies have shown that OMT can help relieve pain and have an impact on various kinematic parameters that could be beneficial to athletes’ health and performance (Licciardone et al., 2005; Brolinson et al., 2012). Performing at high level also requires an optimal interplay of sympathetic and vagal activity (Hedelin et al., 2001; Pagani and Lucini, 2009; Hug et al., 2014). Therefore, our preliminary results might suggest wider opportunities for the use of OMT in athletes, thus opening the way for future randomized controlled trials aimed at testing the effectiveness of OMT as a recovery strategy to restore athletes’ optimal ANS function in the aftermath of a competition and/or during conditions of overtraining syndrome.

## Potential Pathophysiological Links Between OMT and HRV

The specific pathophysiological mechanisms underlying the influence of OMT on ANS function, and specifically HRV, are currently unknown. However, we may put forward several hypotheses. For example, musculoskeletal alterations may increase sympathetic activity via proinflammatory mediators (Pongratz and Straub, 2014), and/or cause a compressive effect on the vagus nerve, given the anatomical relationship of vagal efferents to the musculoskeletal structures at the occiput (Giles et al., 2013). Consequently, the correction of musculoskeletal alterations with OMT may help restore the ANS balance. Moreover, it is becoming increasingly clear that the ANS, particularly the vagus nerve, is involved in the regulation of the inflammatory reflex that controls innate immune responses when tissue is injured or there is a pathogen invasion (Pavlov and Tracey, 2012). The inflammatory reflex has an afferent vagal component that is activated by cytokines and relay information to the hypothalamus. A subsequent efferent signal via the vagus initiates an anti-inflammatory response that prevents the release of inflammatory products into the blood stream (Tracey, 2002). Relatedly, we recently published a meta-analysis demonstrating the presence of a negative relationship between HRV and markers of inflammation (Williams et al., 2019). It is therefore also plausible that the correction of alterations in the musculoskeletal structures the surround the vagus may improve its ability to contribute to anti-inflammatory responses- another way of describing the “structure-function concept” of osteopathy- resulting in increased HRV. However, findings of increased HRV following OMT also in asymptomatic individuals free of injury or somatic dysfunction suggest that other mechanisms may be involved. For example, OMT might directly activate c-tactile fiber afferent projections to brain stem nuclei involved in the regulation of cardiac ANS control. In fact, growing evidence suggests that touch-based interventions such as OMT may play an interoceptive role via c-tactile afferents (McGlone et al., 2017; Edwards et al., 2018; Cerritelli et al., 2020b). It must be noted, however, that in the Edwards’ study (Edwards et al., 2018), changes in interoceptive accuracy following OMT mobilization of the temporomandibular joint were not accompanied by concomitant changes in HRV in healthy subjects. Therefore, future mechanistic research is needed to unveil the precise pathophysiological links between OMT and HRV.

## Conclusion

The study of the relationship between OMT and the ANS is very much in its early days. However, the analysis of HRV in experimental investigations on this link has started to provide preliminary insights into the ability of OMT to tip the ANS balance toward a relatively larger cardiac vagal modulation in healthy subjects. Many questions remain unanswered, the duration of this effect and the specificity of HRV responses to different OMT techniques being perhaps the most critical. We therefore encourage future randomized controlled trials adopting different treatment protocols and longer HRV assessment to address these questions in healthy populations and individuals with diagnosed somatic dysfunctions. Specifically, we would advise researchers to adopt the three Rs design (resting, reactivity, recovery) mentioned in Laborde et al. (2017) for a better understanding of the effects of a single session of OMT on vagally mediated HRV, and also of a multiple-session OMT intervention (Hottenrott et al., 2019). We believe that the results of these trials will provide a more precise quantitative evaluation of the effects of OMT on cardiac ANS function, and will also help identify or refine specific protocols for the use of OMT in conditions characterized by ANS imbalance. In fact, we have presented here preliminary and promising examples of the favorable effects of OMT on HRV in pathological and physiological conditions that are commonly associated with larger sympathetic dominance, namely hypertension, stress exposure, and recovery from sport competition. We are aware that there is still a long road ahead, but, in our view, further substantiation of the influence of OMT on ANS function using HRV will represent the first step toward novel applications of OMT as a preventive and/or complementary strategy in these and potentially other clinical and non-clinical conditions characterized by ANS imbalance. Relatedly, more effort should be directed to the exploration of the mechanisms linking OMT to HRV. Of these, we propose that mechanisms that rely on c-tactile fiber signaling may represent the most promising candidate.

## Data Availability Statement

The raw data supporting the conclusions of this article will be made available by the authors, without undue reservation, to any qualified researcher.

## Ethics Statement

The studies involving human participants were reviewed and approved by the Institutional review board of the Collegio Italiano di Osteopatia in Parma. The patients/participants provided their written informed consent to participate in this study.

## Author Contributions

LC and AS were involved in the conception and design of this article. LC, LL, and MF conducted the pilot study. LC wrote the first draft. LL, MF, and AS revised the manuscript critically for important intellectual content. All authors contributed to the article and approved the submitted version.

## Conflict of Interest

The authors declare that the research was conducted in the absence of any commercial or financial relationships that could be construed as a potential conflict of interest. The reviewer ET declared a past co-authorship with several of the authors LC, AS to the handling Editor
